# Caffeine ameliorates the metabolic syndrome in diet-induced obese mice through regulating the gut microbiota and serum metabolism

**DOI:** 10.1186/s13098-023-00993-3

**Published:** 2023-03-08

**Authors:** Li Chen, Xian-jun Wang, Jie-xin Chen, Jing-cheng Yang, Ling Lin, Xian-Bin Cai, Yong-song Chen

**Affiliations:** 1 Department of Gastroenterology, The First Affiliated Hospital of Shantou University Medical College, Shantou, 515041 Guangdong People’s Republic of China; 2 Department of Endocrinology, The First Affiliated Hospital of Shantou University Medical College, No. 57 Changping Road, Shantou, 515041 Guangdong China; 3 Department of Rheumatology, The First Affiliated Hospital of Shantou University Medical College, Shantou, 515041 Guangdong China

**Keywords:** Caffeine, Gut microbiota, Serum metabolism, Metabolic syndrome

## Abstract

**Objective:**

Obesity is associated with gut microbiota disorders, which has been related to developing metabolic syndromes. The research aims to investigate the effects of caffeine treatment on insulin resistance, intestinal microbiota composition and serum metabolomic changes in high-fat diet (HFD)-induced obesity mice.

**Methods:**

Eight-week-old male C57BL/6 J mice were fed a normal chow diet (NCD) or HFD with or without different concentrations of caffeine. After 12 weeks of treatment, body weight, insulin resistance, serum lipid profiles, gut microbiota and serum metabolomic profiles were assessed.

**Results:**

Caffeine intervention improved the metabolic syndrome in HFD-fed mice, such as serum lipid disorders and insulin resistance. 16S rRNA Sequencing analysis revealed that caffeine increased the relative abundance of *Dubosiella*, *Bifidobacterium* and *Desulfovibrio* and decreased that of *Bacteroides*, *Lactobacillus* and *Lactococcus* to reverse HFD-fed obesity in mice. Additionally, Caffeine Supplementation also altered serum metabolomics, mainly focusing on lipid metabolism, bile acid metabolism and energy metabolism. Caffeine increased its metabolite 1,7-Dimethylxanthine, which was positively correlated with *Dubosiella*.

**Conclusions:**

Caffeine exerts a beneficial effect on insulin resistance in HFD-mice, and the underlying mechanism may be partly related to altered gut microbiota and bile acid metabolism.

## Introduction

Obesity is a serious issue worldwide, and its prevalence continues to rise in most countries [1]. Obesity can be simply summarized as the imbalance between energy intake and expenditure resulting in the accumulation of excess energy in the body, leading to metabolic disorders in the organism [2]. Many kinds of research show it is closely related to type 2 diabetes, non-alcoholic fatty liver disease, and heart diseases [3, 4]. Moreover, Insulin resistance (IR) has long been a problem carried by obesity itself. Although taking diet pills is currently the most popular way to lose weight, they still have some side effects and may improve insulin resistance to a limited extent. For example, Metformin can cause adverse gastrointestinal reactions (5). This is why an urgent search for a functional food that fights obesity while improving insulin resistance is critical.

Coffee and tea are the food products most consumed worldwide [6, 7]. Long-term consumption of tea or coffee has a wide range of health-promoting effects, including anti-obesity [8], prevention of Type II Diabetes [9], as well as inhibition of the development of diabetic complications [10]. Caffeine is the main active ingredient of tea and coffee. A study of green tea containing caffeine suggests that caffeine may be its main anti-obesity ingredient [11]. The beneficial metabolic effects of caffeine on obesity include the downregulation of inflammatory factors expression in the circulation and various tissues, the upregulation of adipocyte lipolysis, the inhibition of lipid synthesis and hepatic steatosis, a reduction in fat mass and the improvement of fatty liver, hepatic/systemic insulin resistance, which have been reported in both humans and animals [12–15]. In addition, in nonobese research models, caffeine was also found to inhibit neonatal cord blood mononuclear cells from releasing TNF-α, improving fructose-induced insulin resistance in mice by enhancing central insulin signaling and Glut4 expression in skeletal muscle to reverse rat ageing-induced insulin resistance [16–18].

Many reports show the gut microbiota has been linked to inflammatory bowel disease [19], obesity [20], autism spectrum disorders [21] and immune system disorders [22] among others. Some components of coffee might have affected the gut microbiota [23–25]. A recent study has shown that EGCG and caffeine, the main component of green tea, can modulate the gut microbiome to fight obesity [26]. Fubrick tea has been confirmed to improve metabolic disorders by regulating intestinal microbiome and caffeine metabolism [27], and the close relationship between gut microbiome and metabolomics suggests that caffeine may affect gut microbiome. What's more, it has been shown that caffeine-induced sleep restriction alters the gut microbiome in mice [28]. However, the relationship between how caffeine affects the gut microbes and its improvement in metabolic syndrome has not been reported. Metabolomics has been widely used because of its potential to help researchers identify the involvement of different biomarkers. Therefore, we focus on investigating whether caffeine, a main component of coffee, ameliorates insulin resistance in diet-induced obesity mice in association with gut microbiota alterations and explores its underlying mechanism through plasma metabolites.

## Method

### Preparation of caffeine solution

Caffeine (Sigma-Aldrich, C0750) was respectively dissolved in sterile water to get a concentration of low-dose caffeine solution (0.5 g/L) and high-dose caffeine solution (1 g/L) for feeding to high-fat mice until mice were sacrificed.

### Animal experiments

Forty male C57BL/6 J mice (Charles River) weighing 20–25 g were bred at Shantou University Medical College (Shantou, China). All animals lived in a specific pathogen-free environment with free access to food and water. After all, mice were acclimatized to the research environment for one week; ten mice were randomly fed with a normal chow diet (NCD group). In contrast, the remaining 30 mice were fed with a 60% high-fat diet (ResearchDiet, D12492) and randomly divided into three groups to fed with the sterile water (HFD group), low-dose caffeine solution (caffeine low-dose group) and high-dose caffeine solution (caffeine high-dose group) at the same time. The ten mice in the same group were randomly labelled and housed in two standard cages (five mice per cage). The body weight of mice and the amount of food intake were measured weekly.

### Biochemical analysis of serum samples

At the end of the experiment, all mice were anaesthetized with sodium pentobarbital (50 mg/kg) intraperitoneally. The eyes were removed to collect the blood samples in 5 mL Vacutainer tubes containing the chelating agent ethylene diamine tetraacetic acid (EDTA). The samples were centrifuged at 4 ℃ for 15 min, and plasma samples were collected and stored at − 80 °C. Serum random blood glucose and lipid profiles such as Total cholesterol (TC), total triglycerides (TG), High-density lipoproteins (HDL) and Low-density lipoproteins (LDL) were measured with an Abbott Architect c16000 instrument (The First Affiliated Hospital of Shantou University Medical College).

### Intraperitoneal glucose tolerance test (IPGTT)

After 12 weeks of caffeine intervention, all mice fasted for 8 h. Using blood glucose test strips, measured the blood glucose level at 0 min. Immediately after that, all animals were intraperitoneally injected with 20% glucose solution(1 g/kg). Subsequently, blood glucose values were respectively measured at 30, 60, 90, and 120 min. Plot the blood glucose-time curve and computer the area under the IPGTT curve (AUC).

### Intraperitoneal insulin tolerance test (IPITT)

The blood glucose values of each animal given adequate diet and water were measured as the blood glucose values at 0 min. Afterwards, blood glucose levels were measured at 30,60,90 and 120 min after giving each mouse an intraperitoneal injection of 0.75U/kg of insulin with fasting without water. The blood glucose-time curve and the AUC of IPITT are similar to that described above.

### 16S rRNA sequencing and data analysis

#### Sequencing

Total genomic DNA from 39 samples was obtained by hexadecyl trimethyl ammonium bromide (CTAB)/sodium dodecyl sulfate (SDS) method, and the concentration and purity of DNA were detected by 1% agarosegels. The DNA was diluted to 1 ng/μl with sterile water. 16S rRNA was amplified using barcode-containing specific primers 341F-806R (V3-V4). After quantifying and identifying PCR products using 1X loading buffer (containing SYBR green) and 2% agarose gel, the mixed PCR products were purified using the AxyPrepDNA Gel Extraction Kit (AXYGEN). Sequencing libraries were generated using the NEB Next^®^ Ultra^™^ DNA Library Prep Kit for Illumina (NEB, USA) according to the manufacturer's recommendations with index codes added. Library quality was evaluated on a Qubit@ 2.0 Fluorometer (Thermo Scientific) and an Agilent Bioanalyzer 2100 system. Finally, the library was sequenced on the Illumina Miseq/HiSeq2500 platform to obtain 250 bp/300 bp paired-end reads.

#### Data analysis

Paired-end reads from raw DNA fragments were merged using FLASH and assigned to each sample based on a unique barcode. Sequence analysis was performed by the UPARSE software package using the UPARSE-OTU and UPARSE-OTUref algorithms. Sequences with ≥ 97% similarity were assigned to the same OTU. We select a representative sequence for each OTU and use the RDP classifier to annotate the taxonomic information of each representative sequence. Histograms of the relative abundance of species at the phylum and genus levels show the community structure of each grouping at different taxonomic levels. Alpha Diversity metrics such as ace and chao1 were used to Analyze Microbiota evenness and diversity. To confirm differences in the taxonomic abundance of individuals between the two groups, STAMP software was used. LEfSe was used to quantify biomarkers within different groups.

### Serum metabolomic analysis

#### Sample extraction

Plasma samples collected as above were thawed at 4 °C. An appropriate amount of sample and pre-cooled methanol/acetonitrile(1:1,v/v) were mixed and centrifuged for 20 min(14000 g, 4 ℃). The supernatant was used for LC-MS/MS Analysis.

#### LC–MS/MS analysis

The serum samples were detected by a UHPLC (1290 Infinity LC, Agilent Technologies). The column temperature, flow rate and injection volume were 25 °C, 0.5 mL/min and 2 μL. The mobile phase consisted of A (water + 25 mM ammonium acetate + 25 mM ammonia) and B (acetonitrile). The gradient elution program was as follows: 0–0.5 min 95%B; 0.5–7 min, B linearly change from 95 to 65%; 7–8 min, B from 65% linear change to 40%; 8–9 min, B maintained at 40%; 9–9.1 min, B linear change from 40 to 95%; 9.1–12 min, B maintained at 95%. Samples are analyzed randomly and continuously to reduce errors caused by fluctuations in the instrument detection signals. The separated samples were analyzed using a quadrupole time-of-flight (AB Sciex TripleTOF 6600) with electrospray ionization in positive and negative ion modes, as described in a previous study [29].

#### Data analysis

The raw data was converted to. mzXML format by ProteoWizard, and imported into the XCMS software for peak alignment, retention time correction and peak area extraction. Subsequently, the data extracted by XCMS were subjected to metabolite structure identification, data pre-processing, data quality evaluation and finally, data analysis. The data acquired were sample normalized, log-transformed (base 10) and auto-scaled on MetaboAnalyst 5.0 (https://www.metaboanalyst.ca/MetaboA-alyst/ModuleView.xhtml). Partial Least Squares—Discriminant Analysis (PLS-DA) and Orthogonal Partial Least Squares Discriminant Analysis (OPLS-DA) are used to perform multivariate statistical analyses. The variable importance in projection (VIP) values showing the contribution of each variable to the classification were measured and counted in the OPLS-DA model. The student's t-test *P* values lower than 0.05 indicated statistically significant differences. Volcano plots further revealed changes in metabolites, with FC > 1.2 being upregulation and FC < 0.8 being downregulation. Lastly, we selected important differential metabolites for metabolite pathway studies in the KEGG (Kyoto Encyclopedia of Genes and Genomes, http://www.kegg.jp/) database.

### Omics association analysis

The relationships between different bacterial lineages and metabolites were obtained by correlation analysis using Spearman’s correlation method for the critical differential metabolites screened by metabolomics and Intestinal microbes that differed significantly at the genus level by 16S sequencing. Correlation heatmap and hierarchical clustering analysis were performed on R language (4.1.2) software.

### Statistical Analysis

All data are expressed as mean ± SEM. Statistical analysis between the control and experiment groups was performed using One-way ANOVA on SPSS 25 software. The Bonferroni post hoc test and the Dunnett T3 post hoc were respectively used for conformity to chi-square and non-conformity chi-square. *P*-value < 0.05 was considered statistically significant. Graphs were made using GraphPad Prism 8 software.

## Result

### Caffeine mitigated metabolic disorders in mice fed with a high-fat diet

**Fig. 1:**
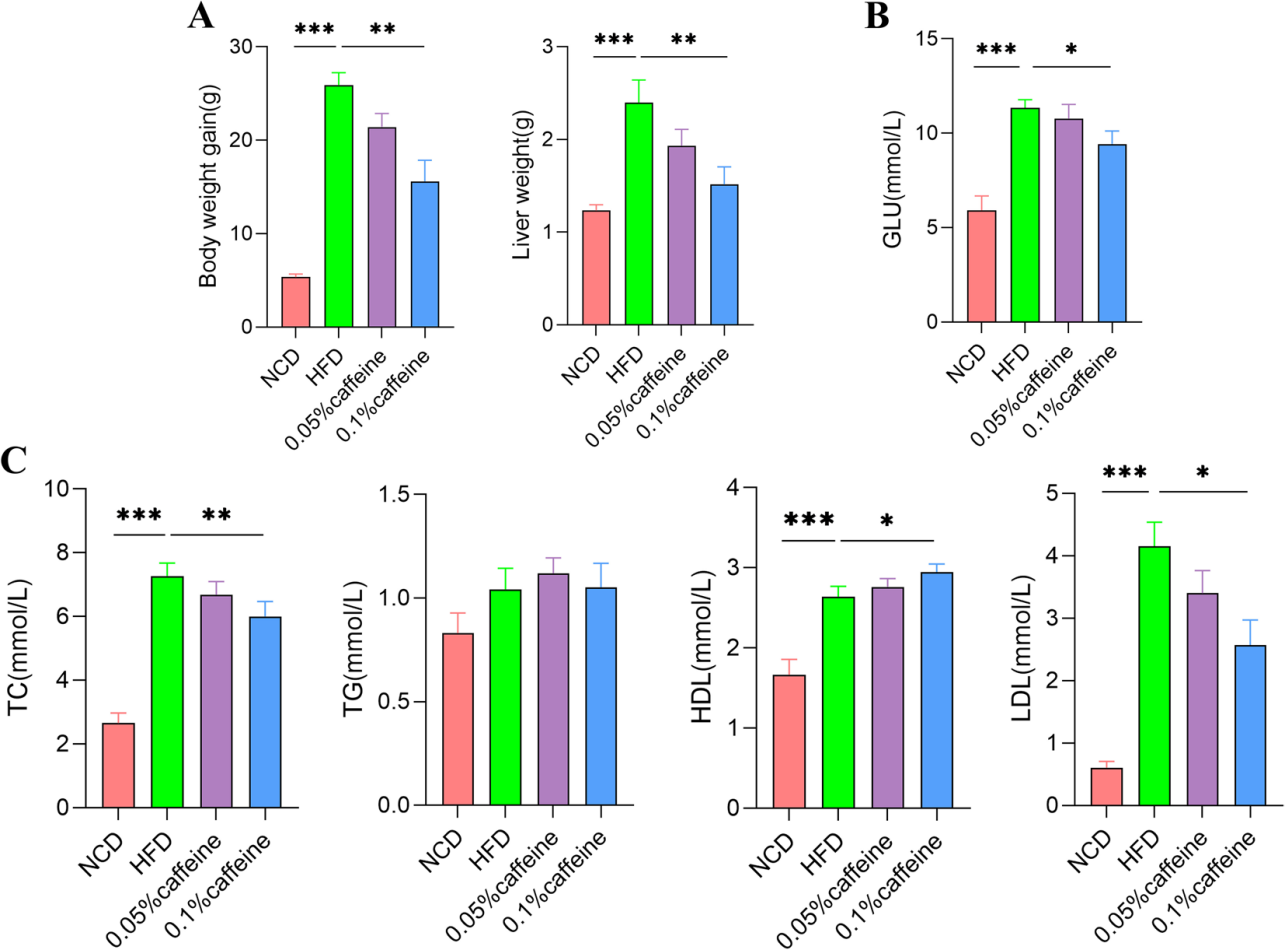
Caffeine alleviated the Hyperlipidemia in the HFD-mice. (A) Caffeine high-dose treatment decreased the body weight and liver weight in obese mice. (B) Caffeine high-dose significantly improved Hyperlipidemia compared with the HFD group. c Caffeine high-dose intervention decreased random blood sugar in obese mice. NCD, HFD, caffeine low-dose and caffeine high-dose (n == 9/10 per group) groups. Data are presented as the mean± SEM. *p < 0.05; **p < 0.0l;***p < 0.001 (HFD group versus NCD group, caffeine low-dose group, caffeine high-dose group)

**Fig. 2:**
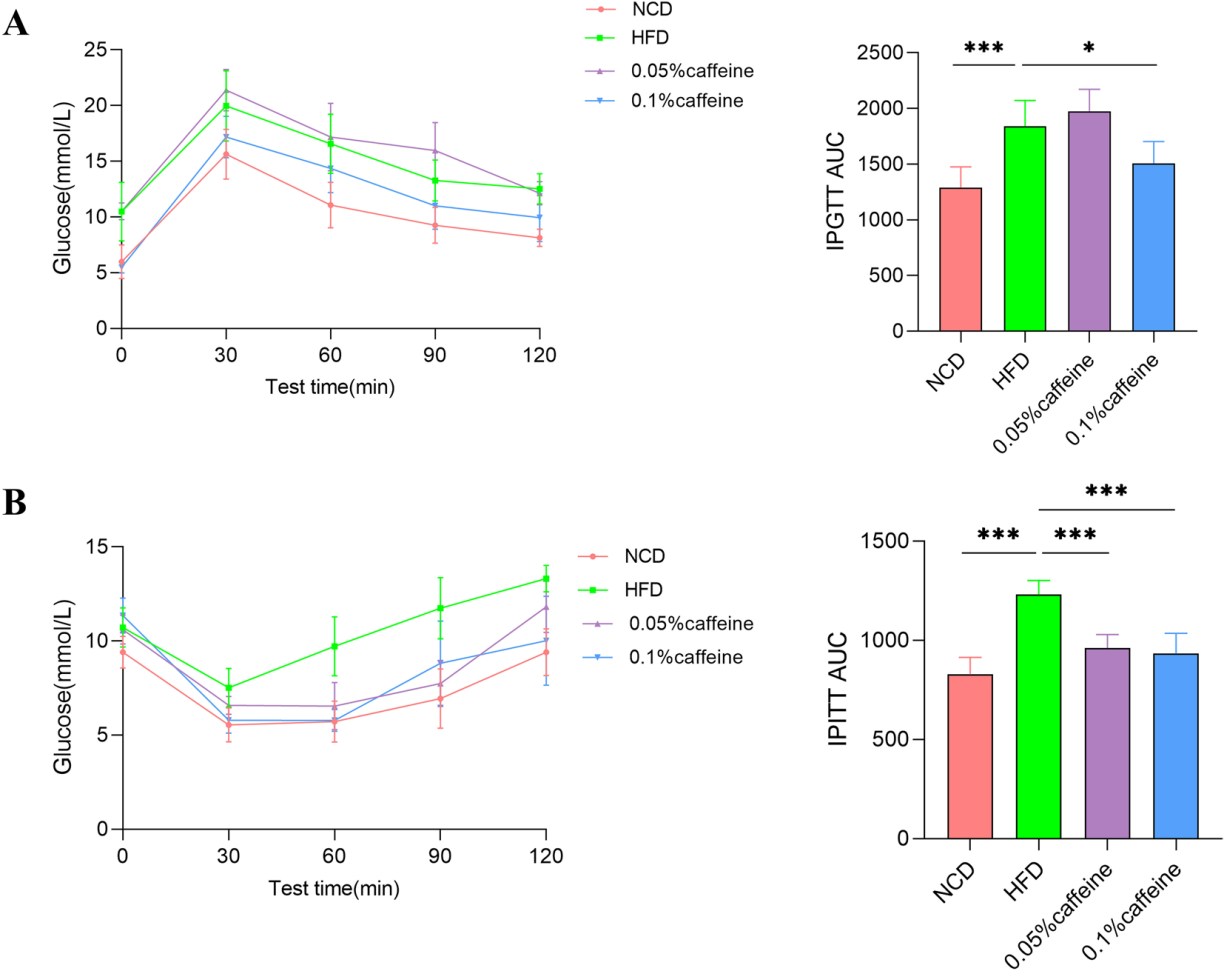
The AUC of IPGTT and IPITT were respectively decreased in insulin resistance mice after caffeine treatment. NCD, HFD, caffeine low-dose and caffeine high-dose (n == 5 per group) groups

Metabolic syndrome is characterized by obesity, diabetes, impaired glucose regulation, and dyslipidemia. As shown in Fig. 1A, B, body weight gain and liver weight were increased in HFD mice in comparison with NCD mice(*p* < 0.001), while caffeine high-dose treatment significantly reduced these indices in comparison with HFD mice(*p* < 0.01). Compared with the NCD mice, serum triglyceride (TG), total cholesterol (TC) and low-density lipoprotein cholesterol (LDL-C) levels were elevated. However, the high-density lipoprotein cholesterol (HDL-C) level was also increased(*p* < 0.001). Consequently, caffeine high-dose significantly downregulated TC and LDL-C levels and upregulated HDL-C levels (Fig. 1C, p < 0.01; *p* < 0.05). Similarly, caffeine high-dose reduced plasma random blood glucose in HFD mice (Fig. 1B, p < 0.05). IPGTT and IPITT tested insulin resistance. Our results indicated that supplementation with high caffeine dose rather than a low dose decreased the AUC of GTT and ITT curve, increasing HFD in mice (Fig. 2;* p* < 0.001; *p* < 0.05). Overall, caffeine high-dose improved the metabolic disorders associated with high-fat diet-induced obesity.

### Caffeine remodeled the gut microbiota in HFD mice

**Fig. 3:**
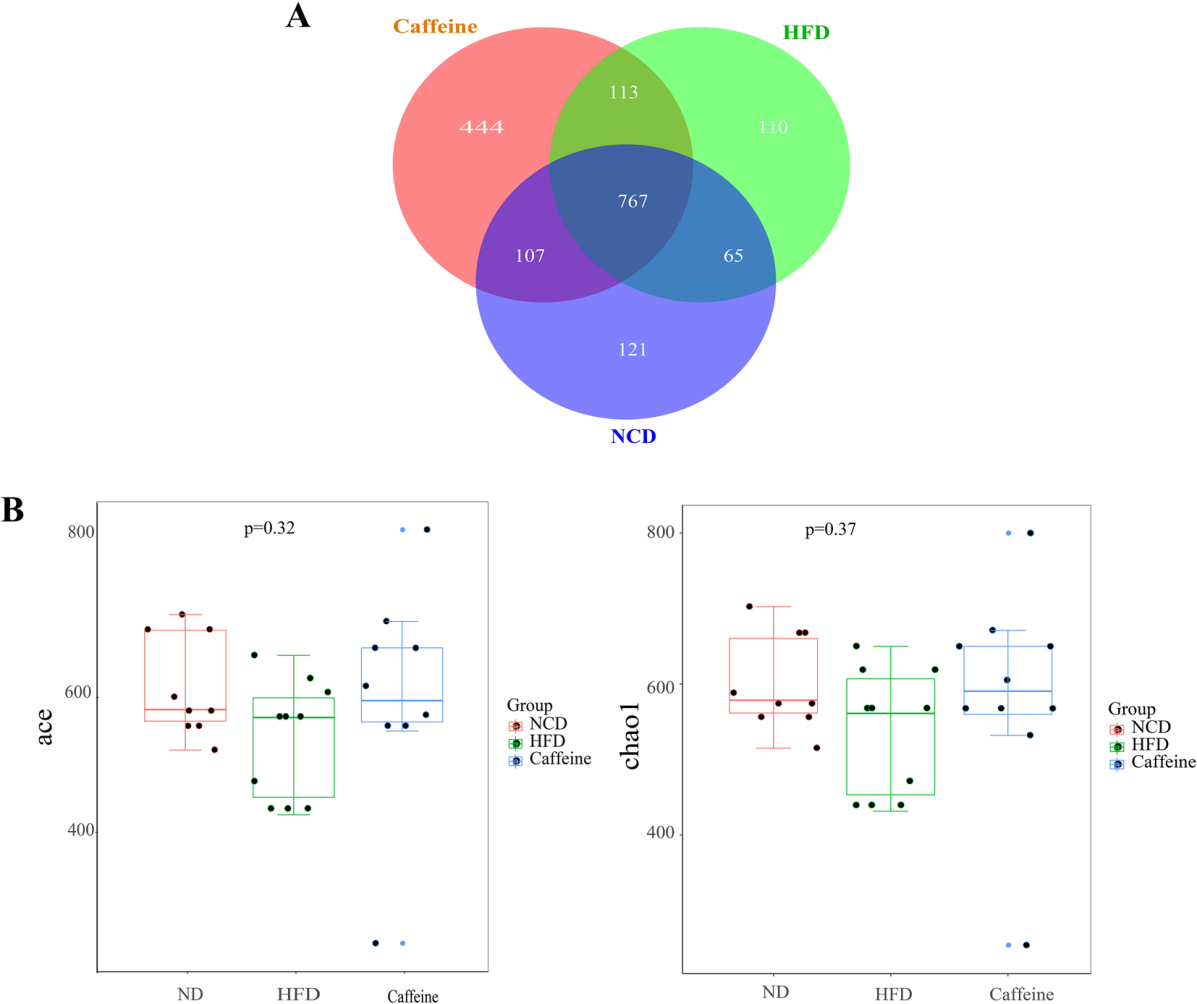
Caffeine treatment altered the number and Alpha diversity of gut microbiota in HFD-mice. (A) A Venn diagram in the three groups. (B) Ace and chaol indexes were higher in the caffeine high-dose group than in the HFD group

**Fig. 4:**
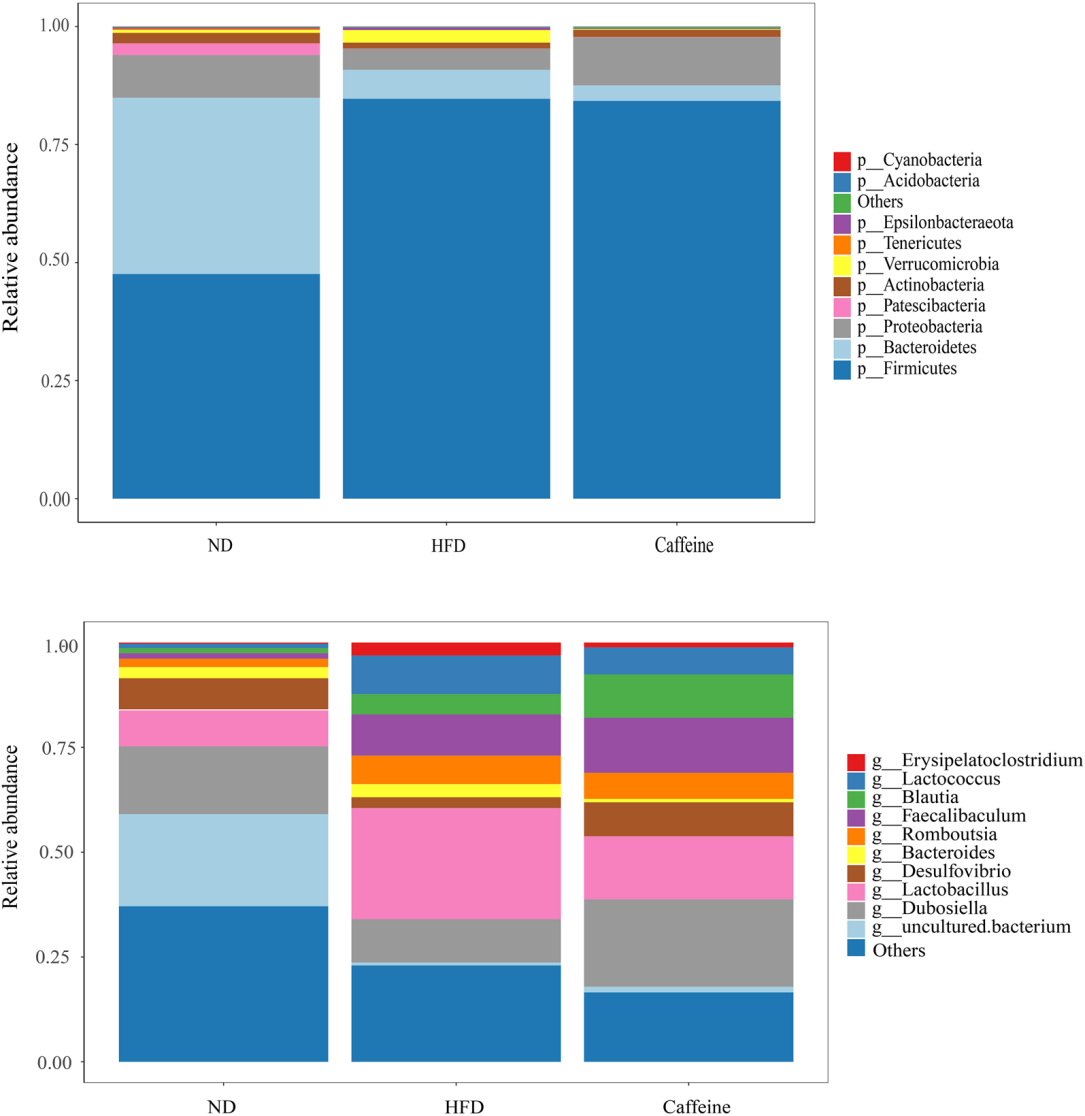
Caffeine high-dose treatment altered the relative abundances of some gut microbiota at the phylum and genus level

**Fig. 5:**
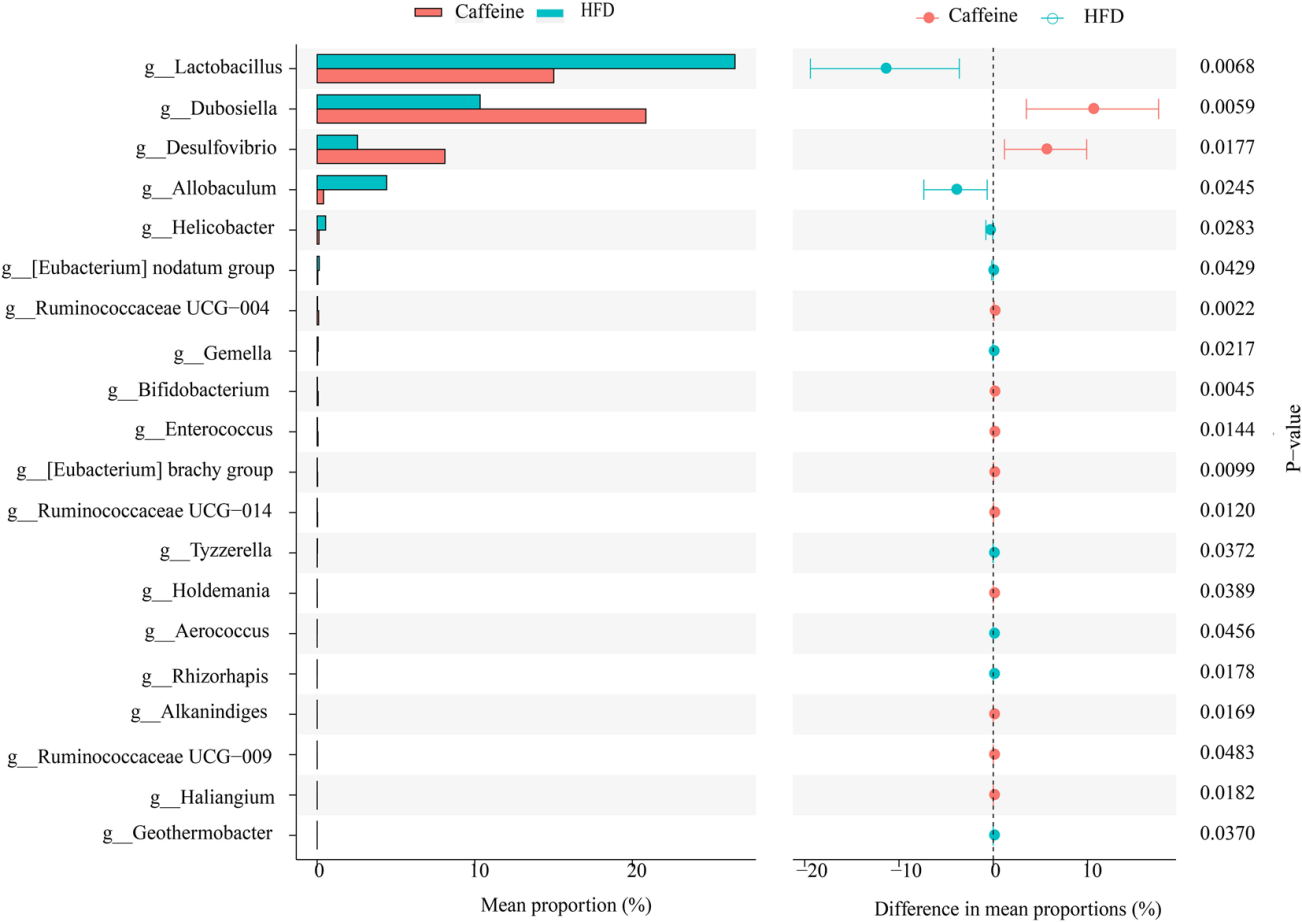
The welch t-test of gut microbiota between HFD and caffeine high-does group

**Fig. 6:**
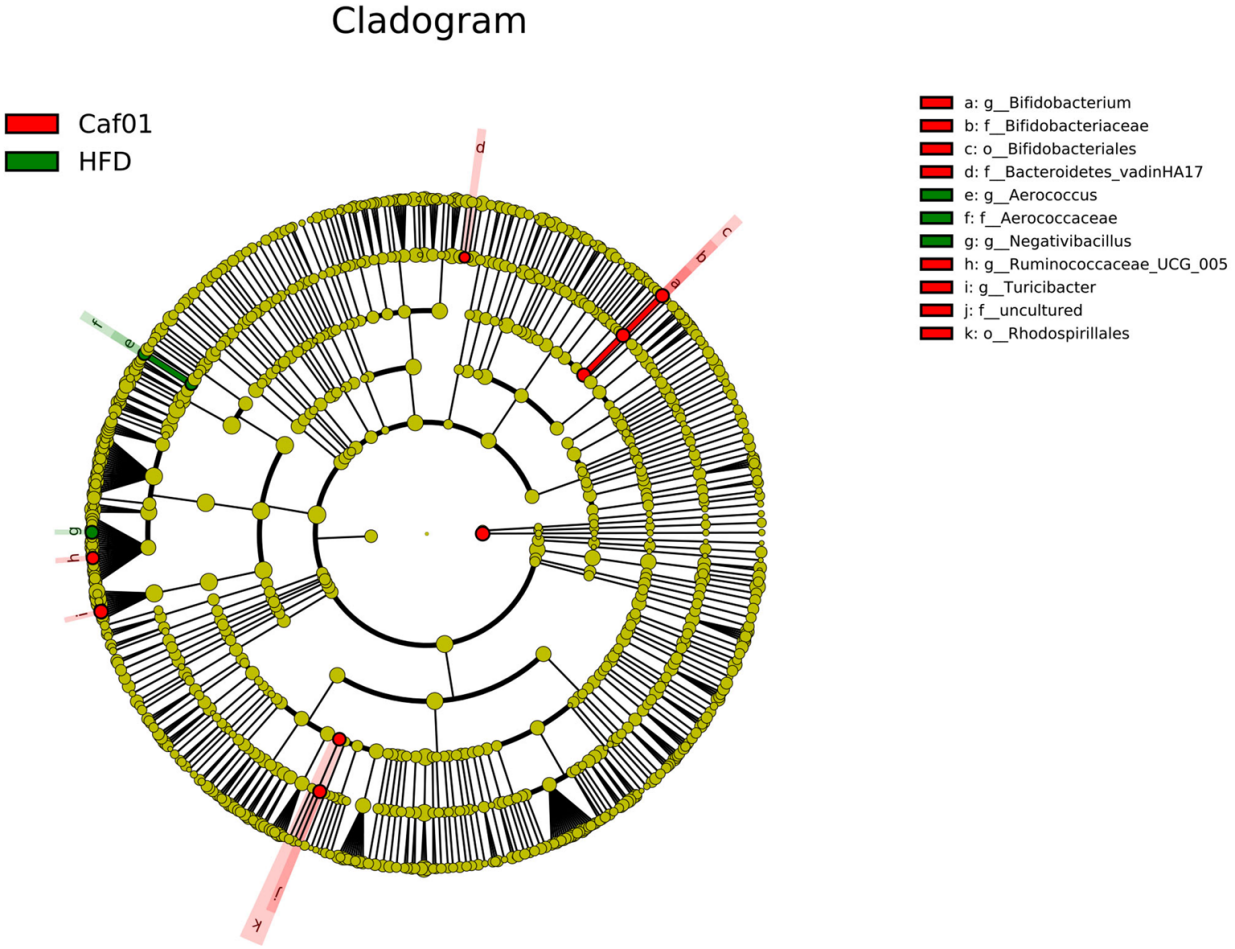
LEfSe analysis identified the most differentially abundant taxa of gut microbiota between the HFD group and caffeine high-dose (n = 9/10 per group) groups

**Fig. 7:**
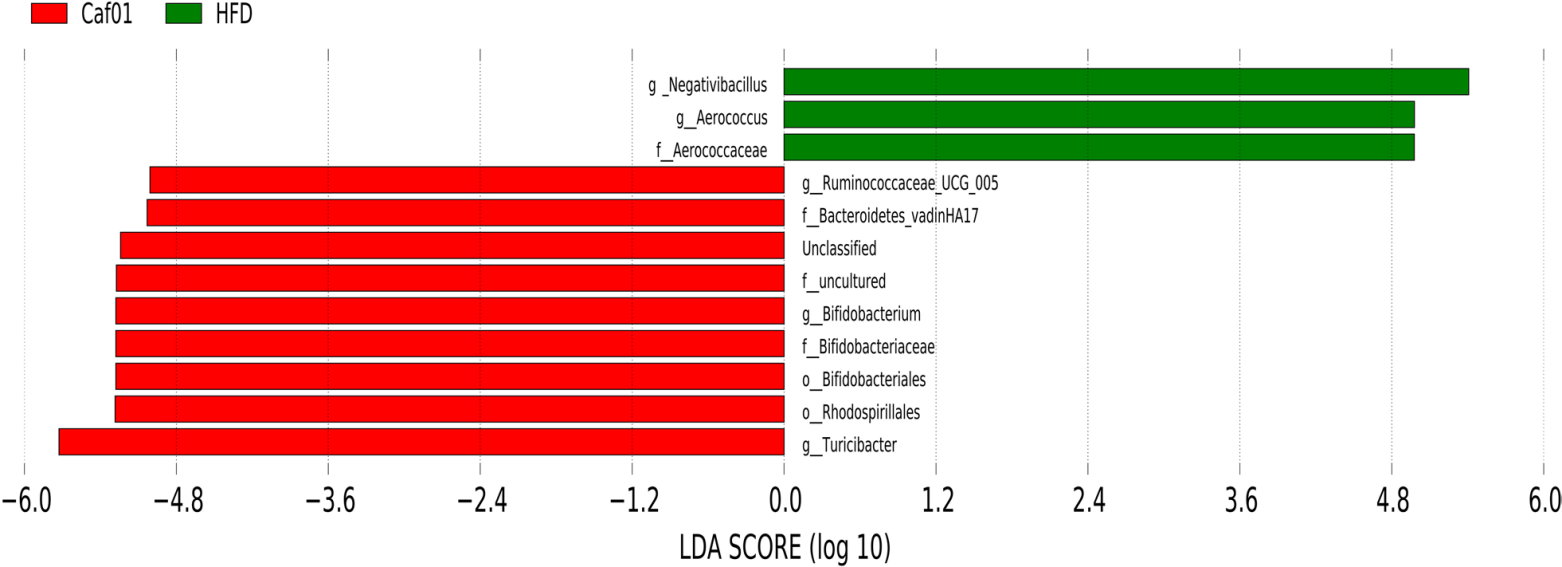
LDA scores ofLEfSe analysis> 4 are shown

Growing evidence indicates Gut microbiota dysbiosis has been repeatedly seen in insulin resistance. To investigate whether caffeine affects the gut microbiota, we collected faeces at 12 weeks of NCD, HFD and caffeine high-dose + HFD mice and then profiled the microbiota composition by 16S rRNA gene sequencing. We performed Splicing, filtering, and chimaera removal on 100897 raw tags from 29 faecal samples to get 83789 clean sequences clustered into 1821 operational taxonomic units (OTUs) with consistently higher than 97% for further analysis. As observed on a Venn diagram (Fig. 3A), 767 OUT were shared among the three groups, and each group had unique OTUs. Ace and chao1, representative Alpha analyses indices (Fig. 3B), showed HFD decreased microbiota species richness and diversity, while caffeine seemed to reverse this change (with no statistical significance). As expected, *Firmicutes* was the most abundant phylum of all samples; however, caffeine seems not to affect *Firmicutes*. We observed caffeine decreased further *Bacteroidetes*, which may be associated with a reduction in *Bacteroides* at the genus level (Fig. 4). Among the significant microbial communities at the genus level, the relative abundance of *Dubosiella* and *Desulfovibrio* was decreased by HFD mice compared with the NCD mice, while caffeine increased these two microbiotas. Moreover, it also raised *Faecalibaculum* and *Blautia*. At the same time, caffeine decreased the relative abundance of *Lactobacillus*, *Romboutsia*, *Lactococcus* and *Erysipelatoclostridium* increased by HFD mice (Fig. 4). We did a Welch t-test with an abundance index for the HFD and caffeine high-dose groups to further explore whether caffeine affected the faecal microbiota. We found 19 OTUs significantly different (*p* < 0.05). As shown in Fig. 5, we found the differences between *Dubosiella*, *Desulfovibrio* and *Lactobacillus* were statistically significant. Furthermore, caffeine elevated *Ruminococcaceae UCG-004*, *Bifidobacterium*, *[Eubacterium] brachy group*, *Ruminococcaceae UGC-014*, *Enterococcus*, *Alkanindiges*, *Haliangium*, *Holdemania* and *Ruminococcaceae UCG-009* and decreased *Gemella*, *Allobaculum*, *Helicobacter*, *Geothermobacter*, *Tyzzerella*, *[Eubacterium] no datum group* and *Aerococcus*. Similarly, we used LDA Effect Size (LEfSe) analysis to identify communities or species that significantly impacted the sample delineation shown in Fig. 6. Species with LDA values greater than 4 were set as statistically different markers between groups (Fig. 7).

### Caffeine changes plasma metabolites in high-fat diet mice

**Fig. 8:**
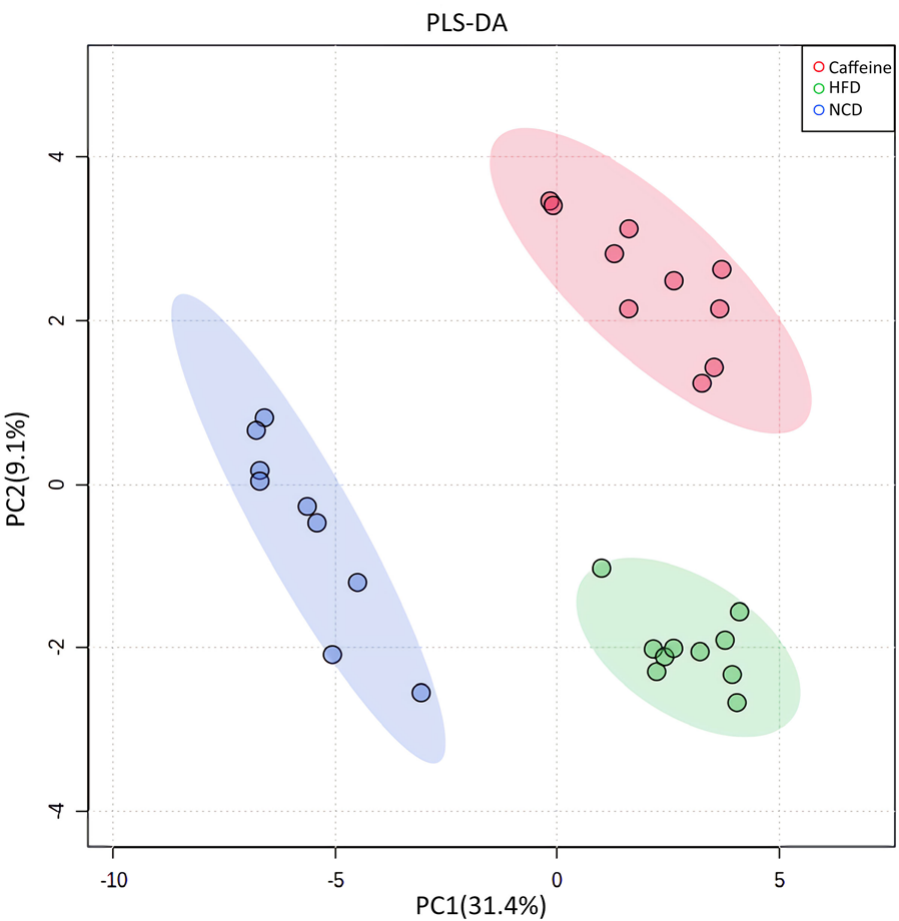
PLS-DA scores of serum metabolites in high-dose groups of NCD, HFD and caffeine high-dose group

**Fig. 9:**
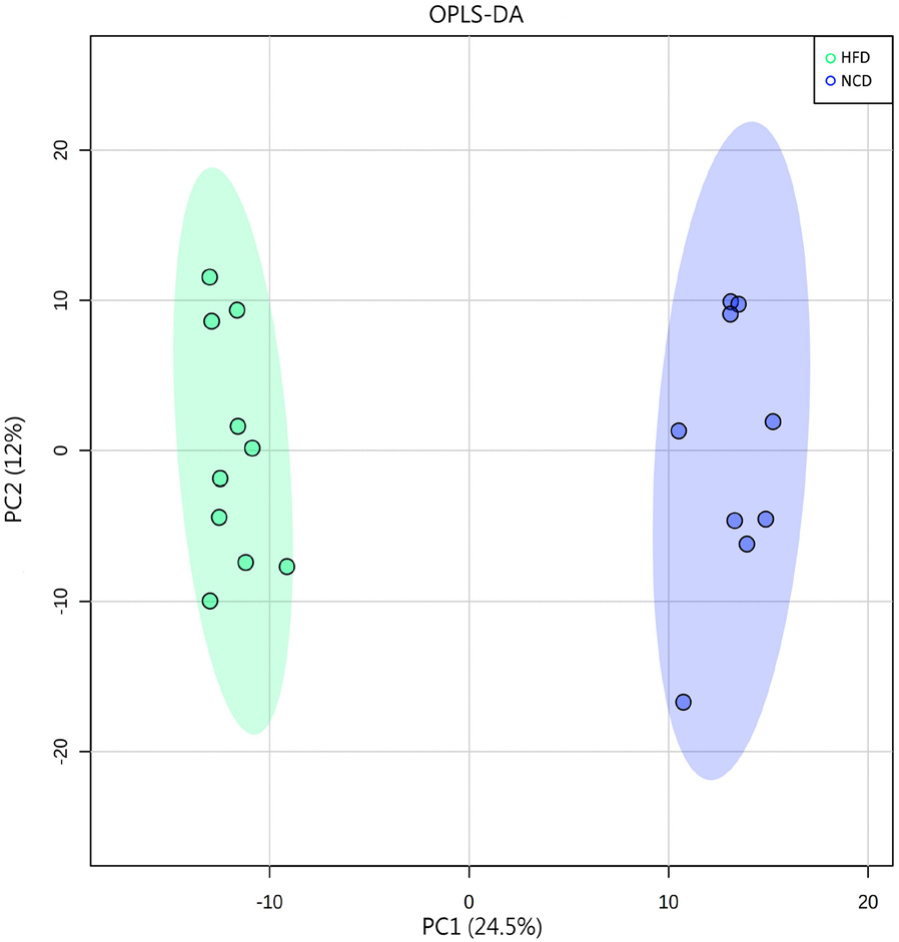
Scores plots of OPLS-DA of serum metabolites between NCD and HFD groups

**Fig. 10:**
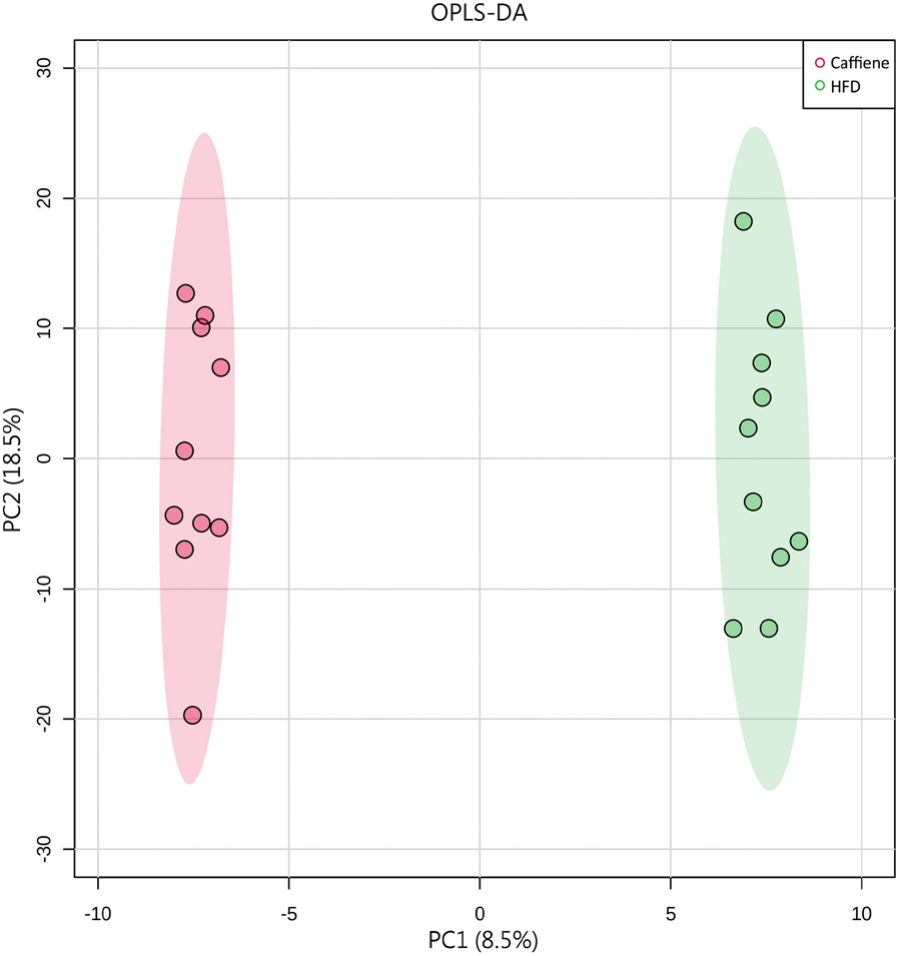
Scores plots of OPLS-DA of serum metabolites between Caffiene and HFD groups

**Fig. 11:**
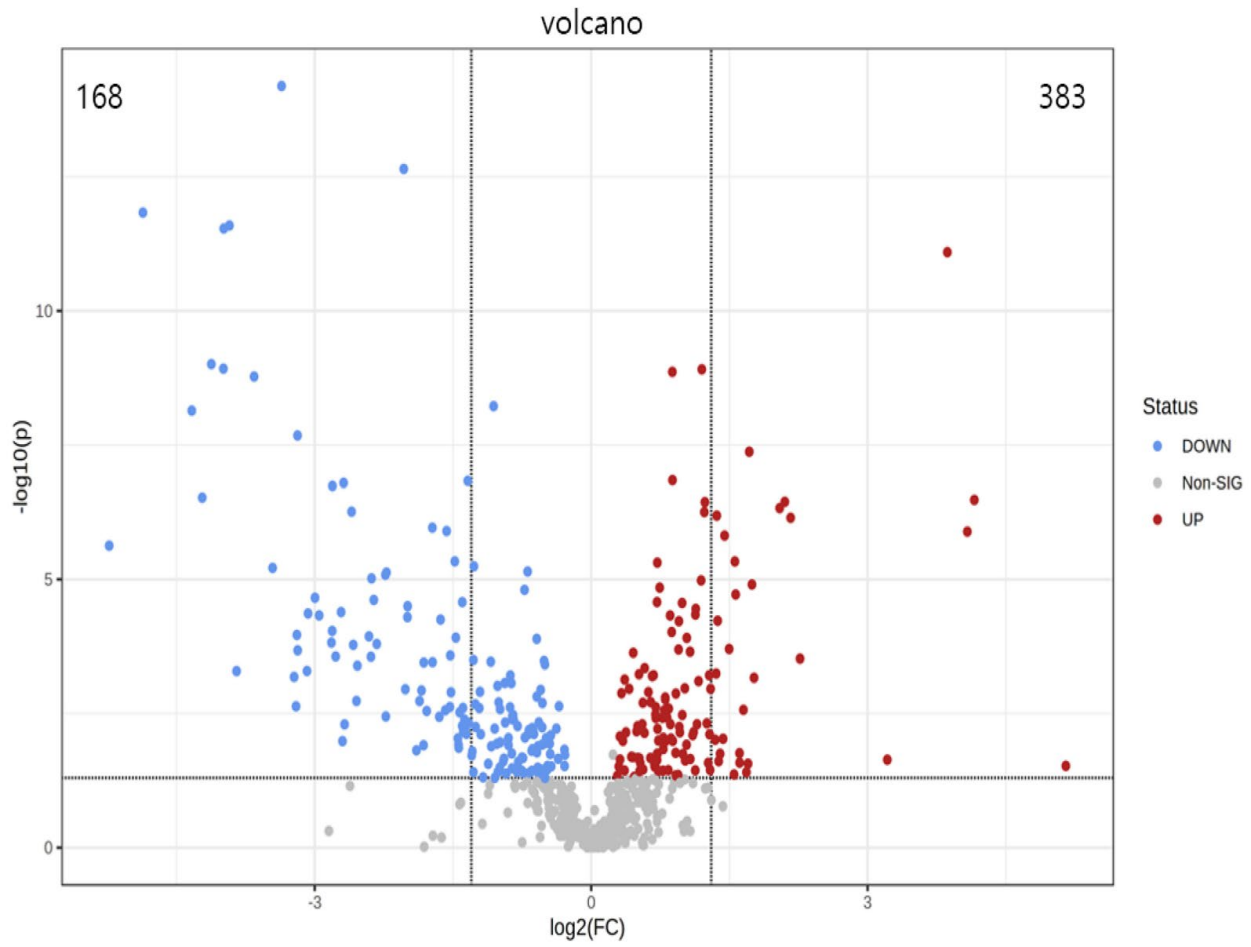
The volcano plot of serum metabolites between NCD and HFD groups

**Fig. 12:**
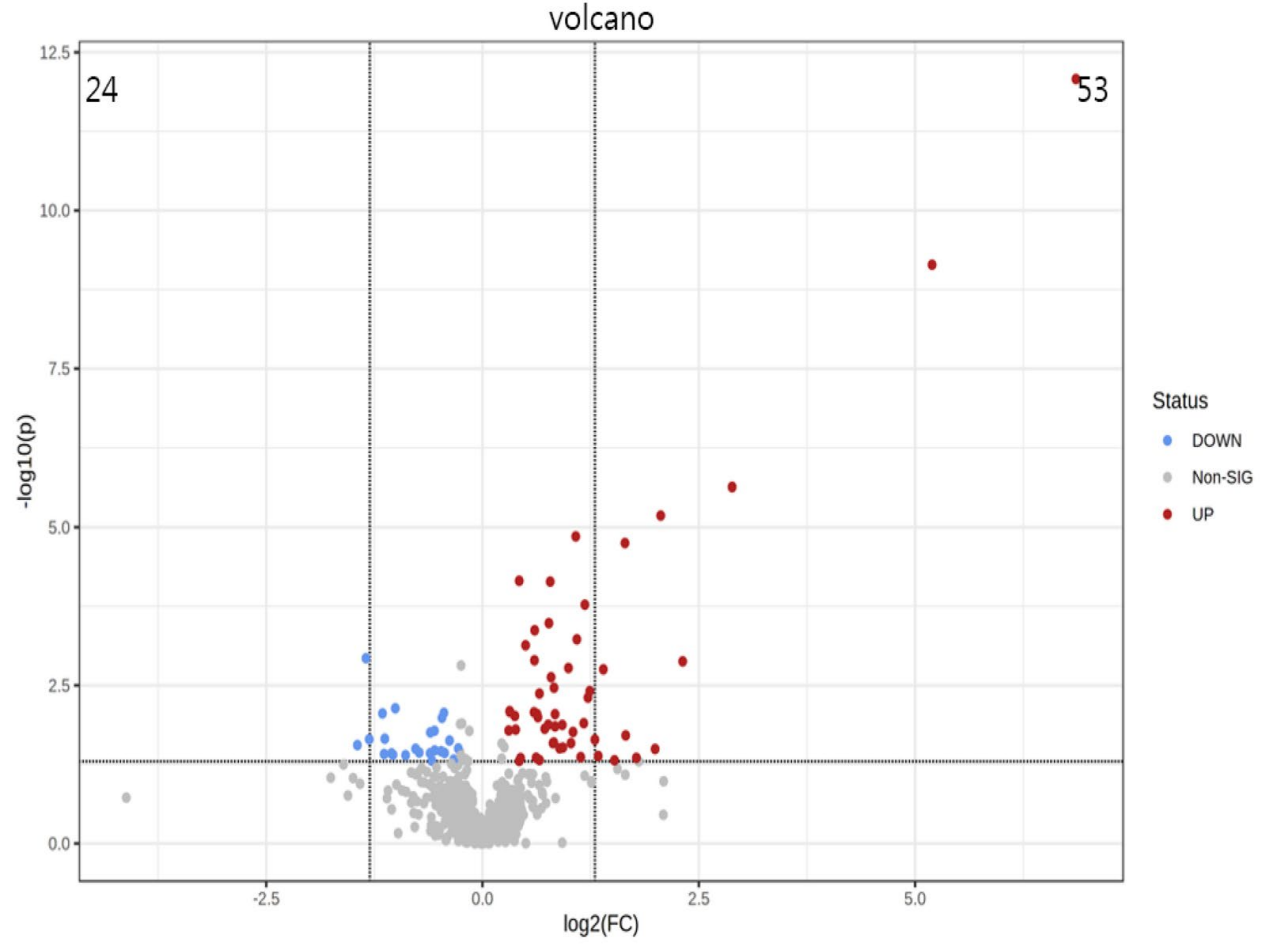
The volcano plot of serum metabolites between Caffiene and HFD groups

PCA is a statistical method that can predict the species with the most enormous difference between groups, which is an unsupervised model; while PLS-DA is a supervised model, which can maximize the difference between groups according to a predefined classification, which has better separation than PCA. The PLS-DA plot showed that there was a clear separation between NCD and HFD and caffeine high-dose groups (Fig. 8). To identify differential metabolites, the OPLS-DA and its corresponding variable importance of projection (VIP) were applied. Consistent with the PLS-DA results, the OPLS-DA model also showed NCD group could be well distinguished from the HFD group, and significant distinctions were observed between HFD and caffeine high-dose group (Figs. 9, 10). In addition, compared to the NCD group, the volcano plot showed that 168 metabolites were downregulated and 383 metabolites were upregulated under high-fat diet induction; caffeine high-dose in obese mice resulted in 24 metabolites downregulated and 53 metabolites upregulated in serum (Figs. 11, 12).

**Fig. 13:**
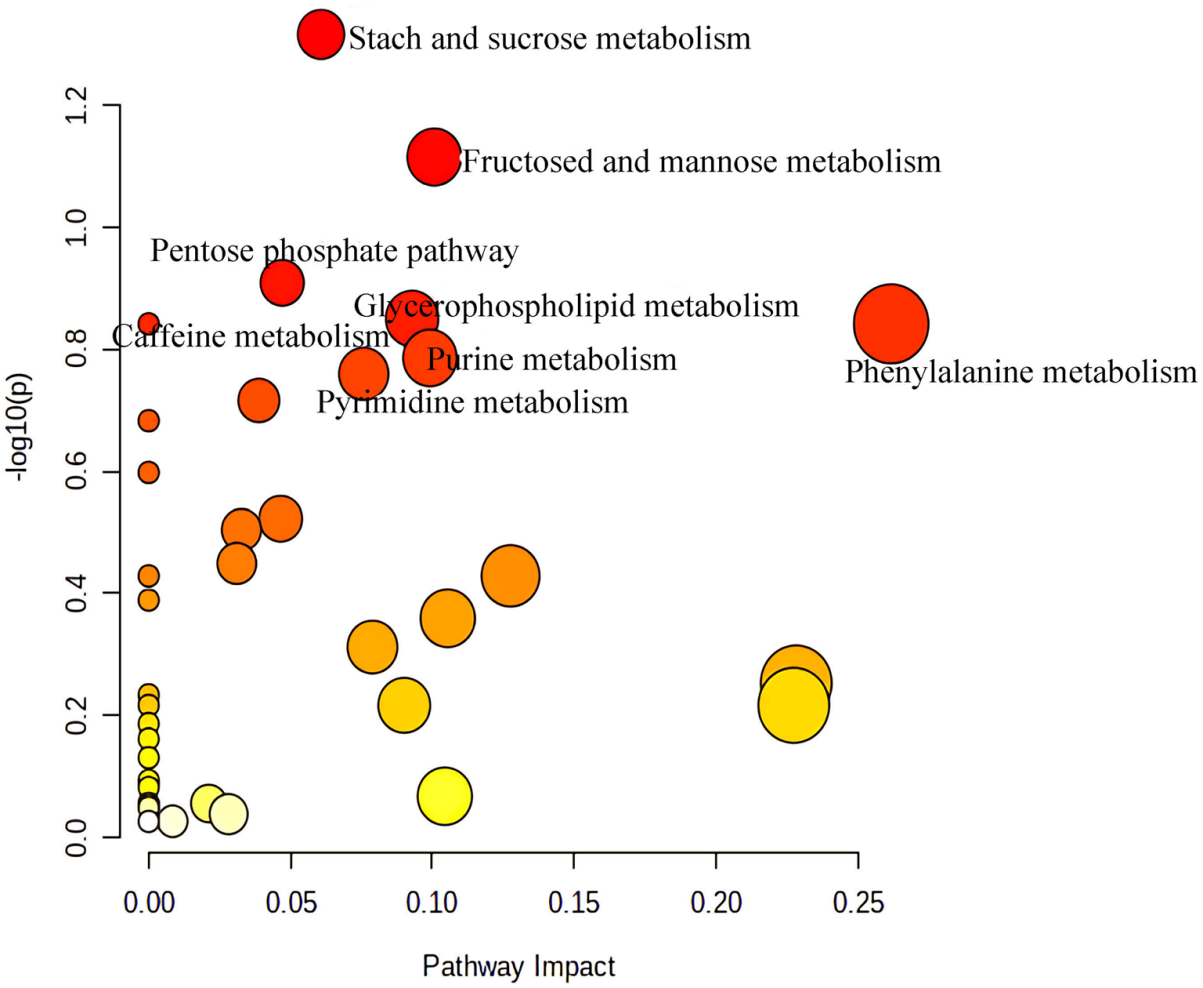
Summary of pathway analysis of serum samples between NCD and HFD group

**Fig. 14:**
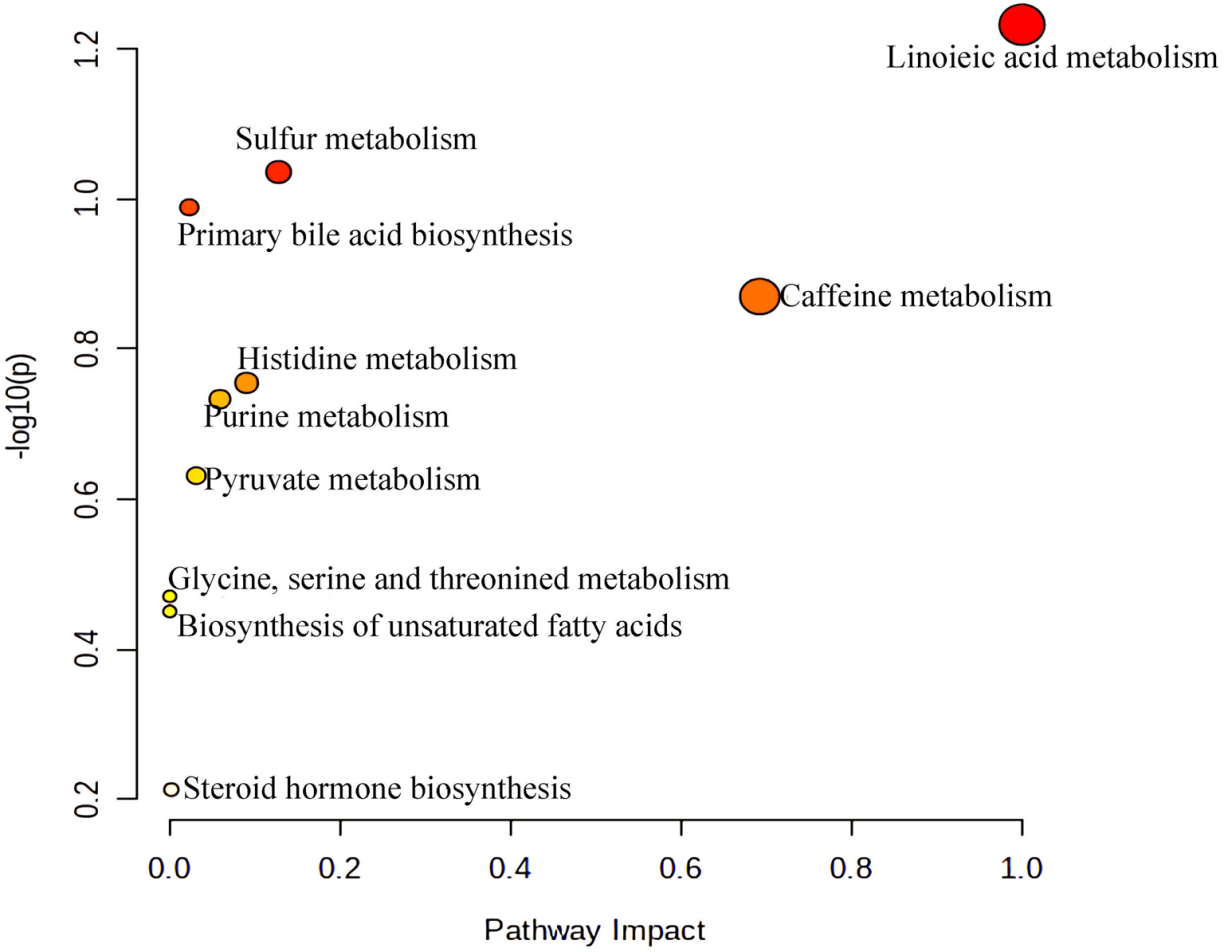
Summary of pathway analysis of serum samples between between HFD and caffeine high-dose group

Next, we used differential metabolites (FC > 1.2 or FC < 0.8, *p* < 0.05, VIP > 1.0) for metabolic pathway screening. Compared with the NCD group, the top 8 KEGG pathways that changed in the HFD group mainly included Starch and sucrose metabolism, Fructose and mannose metabolism, Pentose phosphate pathway, Glycerophospholipid metabolism and caffeine metabolism, which focuses on energy metabolism(Fig. 13); while the pathways changed by caffeine high-dose mainly included Linoleic acid metabolism, Sulfur metabolism, Primary bile acid biosynthesis, Caffeine metabolism, Histidine metabolism, Purine metabolism, Pyruvate metabolism, Glycine, serine and threonine metabolism, Biosynthesis of unsaturated fatty acids and Steroid hormone biosynthesis could be seen(Fig. 14). The Specific trends of variation of differential metabolites contained in metabolic pathways are shown in Table 1.

**Table 1:** The differential metabolites in the pathway after caffeine treatment

Metabolites	VIP		FC		Trend		Pathway
	N v H	H v C	N v H	H v C	N v H	H v C	
Linoleic acid	–	1.56	–	1.34	–	↑*	a, i
Adenosine 5′- phosphosulfate	1.33	1.94	0.5	1.89	↑##	↑*	b, f
Adenosine 5′- monophosphate	1.26	1.94	0.52	1.79	↓##	↑*	f
Pyruvaldehyde	1.09	1.55	2.41	0.49	↑#	↓*	g
Cholic acid	–	1.99	–	0.45	–	↓***	c
Glycocholic acid	1.04	1.99	0.53	1.55	↓#	↑**	c
1,7-Dimethylxanthine	-	3.34	-	116.04	-	↑***	d
N-methylhistamine	1.07	1.73	1.37	0.77	↑#	↓*	h
22R-Hydroxycholesterol	1.24	1.66	0.39	1.9	↓##	↑*	j

NCD, HFD and caffeine high-dose (n = 9/10 per group) groups. #*p* < 0.05 as compared to NCD group; ##*p* < 0.01 as compared to NCD group; ##*p* < 0.001 as compared to NCD group; *p < 0.05 as compared to HFD group; **p < 0.01 as compared to HFD group; ***p < 0.001 as compared to HFD group; ↑, content increased; ↓, content decreased; vs, versus; N, NCD group; H, HFD group; C, caffeine high-dose; VIP, variable importance of projection; FC, fold change. a: Linoleic acid metabolism; b: Sulfur metabolism; c: Primary bile acid biosynthesis; d: Caffeine metabolism; e: Histidine metabolism; f: Purine metabolism; g: Pyruvate metabolism; h: Glycine, serine and threonine metabolism; i: Biosynthesis of unsaturated fatty acids; j: Steroid hormone biosynthesis

### Correlation analysis of gut microbiota, serum untargeted metabolites, physiological data and insulin resistance indicators

**Fig. 15:**
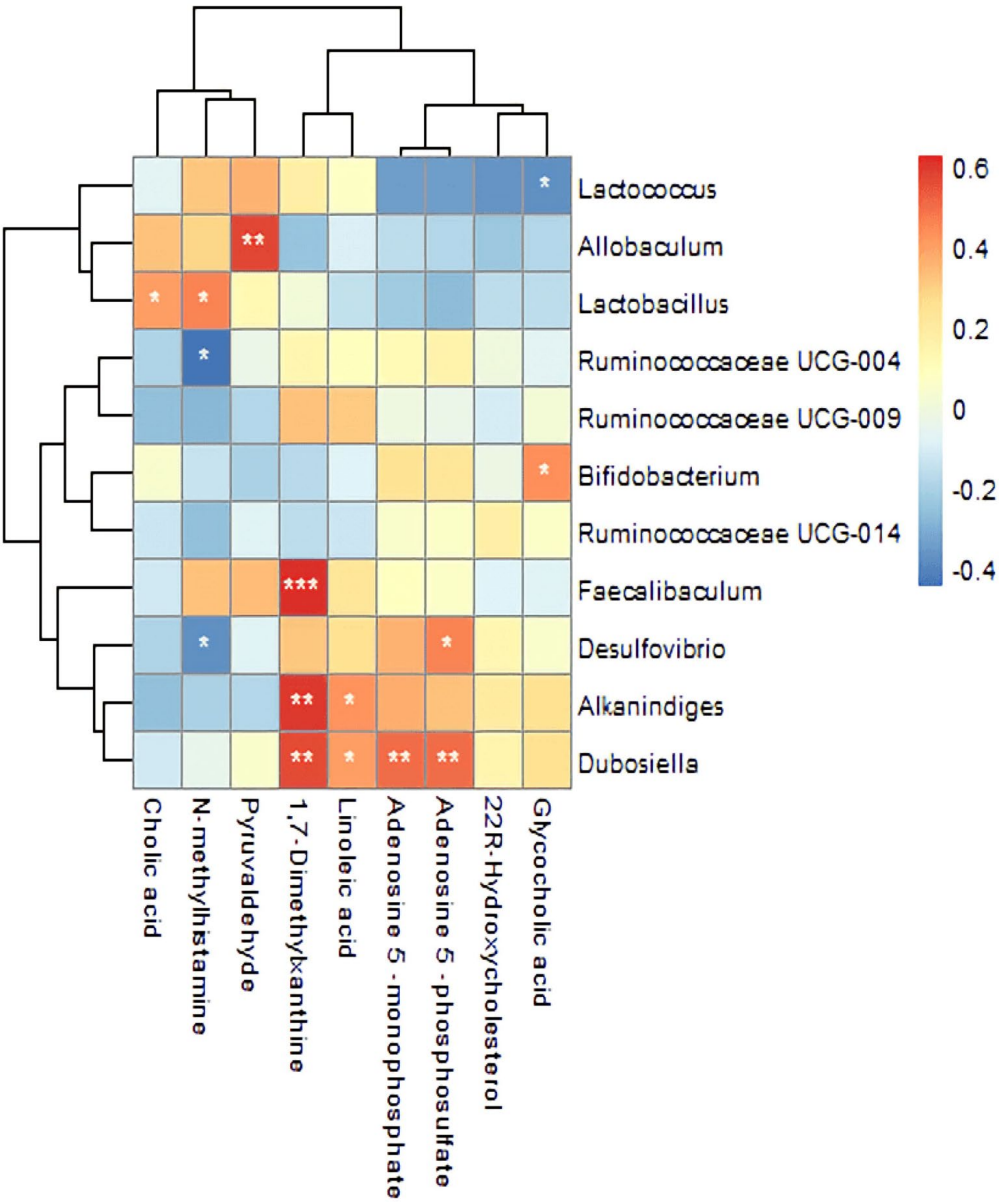
Correlation Analysis of Gut Microbiota and Serum Metabolomics. The colors of grids indicate the correlation analysis value of spearman's correlation analysis. Grids in red represent positive correlations (correlation analysis value more than 0.1), while grids in blue represent negative correlations (correlation analysis value less than -0.1). Color coding scale represents the correlation analysis value from heatmap, the deeper red or blue indicates higher correlation values. *p< 0.05, **p< 0.01, ***p< 0.001

**Fig. 16:**
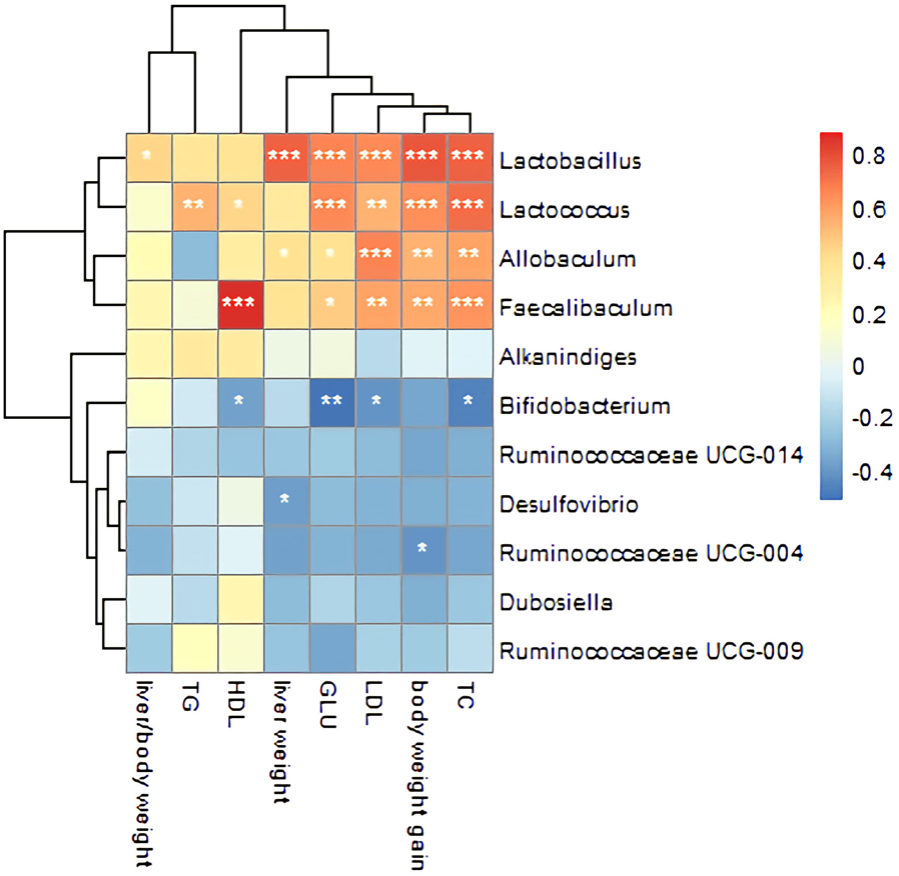
Correlation Analysis of Gut Microbiota and Physiological data. The colors of grids indicate the correlation analysis value of spearman's correlation analysis. Grids in red represent positive correlations (correlation analysis value more than 0.1), while grids in blue represent negative correlations (correlation analysis value less than -0.1). Color coding scale represents the correlation analysis value from heatmap, the deeper red or blue indicates higher correlation values. *p< 0.05, **p< 0.01, ***p< 0.001

**Fig. 17:**
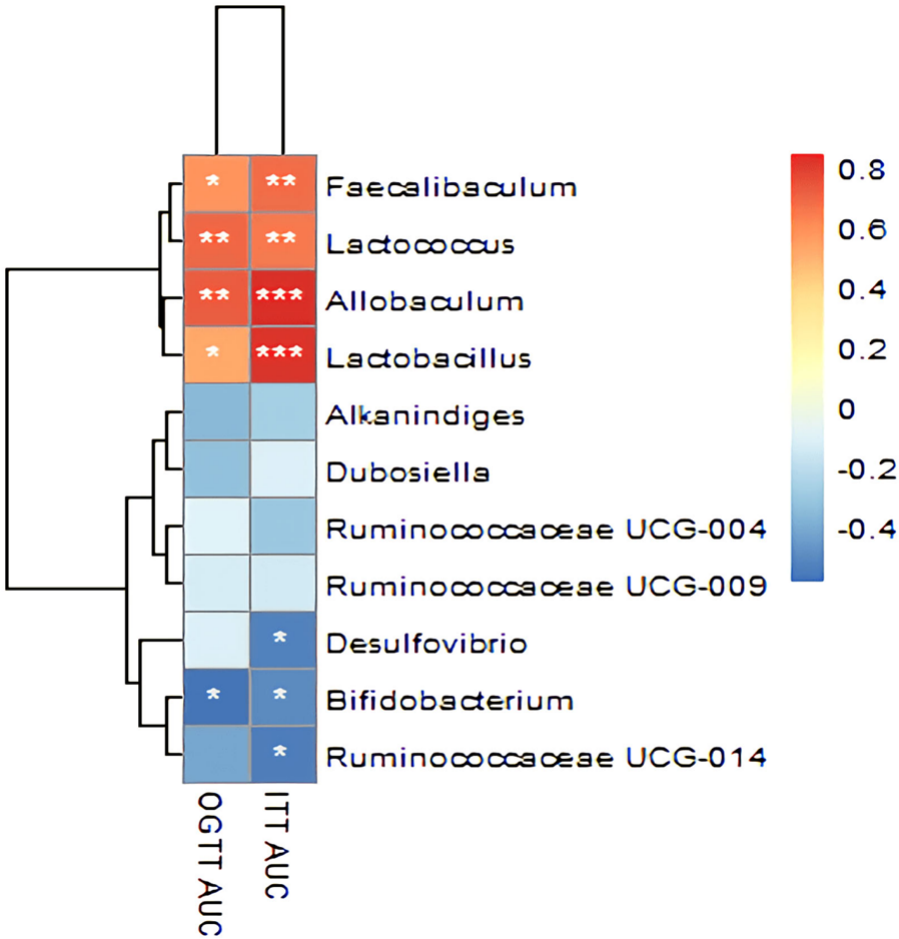
Correlation Analysis of Gut Microbiota and Insulin resistance indicator. The Y-axis indicates the different abundance of the gut microbiota. The colors of grids indicate the correlation analysis value of spearman's correlation analysis. Grids in red represent positive correlations (correlation analysis value more than 0.1), while grids in blue represent negative correlations (correlation analysis value less than -0.1). Color coding scale represents the correlation analysis value from heatmap, the deeper red or blue indicates higher correlation values. *p< 0.05, **p< 0.01, ***p< 0.001

Correlation analysis of serum untargeted metabolites and gut microbiota at genus level in the NCD, HFD and caffeine high-dose groups was presented by correlation heatmap. As shown in Fig. 15, caffeine metabolomics such as 1,7-Dimethylxanthine showed positive correlations with *Dubosiella*, *Alkaninndiges* and *Faecalibaculum*. Pyruvaldehype was positive correlations with *Allobaculum*. In addition, *Desulfovibrio*, *Dubosiella*, and *Alkaninndiges* correlated positively with most of the metabolites. N-methylhistamine and Cholic acid showed negative correlations with some gut microbiota. In addition, *Bifidobacterium*, *Desulfovibrio* and *Ruminococcaceae* showed negative correlations with and *Lactobacillus*, *Lactococcus*, *Faecalibaculum*, and *Allobaculum* showed positive correlations with physiological data and insulin resistance indicators (Figs. 16, 17).

## Discussion

In our study, we found that caffeine high-dose could reduce body weight and liver weight, and improve hyperlipidemia and insulin resistance in the HFD mice, demonstrating caffeine has a therapeutic effect on metabolic syndromes, which is consistent with previous studies [15–17]. Moreover, we elucidate the mechanism of this anti- metabolic syndrome, which may involve gut microbiota, bile acid metabolism, lipid metabolism, and energy metabolism.

The nature of the intestinal microbiome has been shown to be closely related to IR in several human and animal model studies [30, 31]. 16S rRNA sequencing was used to investigate how caffeine affects the composition of the gut microbiome. Compared to the NCD group, HFD mice have a substantial reduction in the abundance of *Bacteroidetes* and a proportional increase in *Firmicutes*, similar to previous studies [20]. However, caffeine further reduced *Bacteroidetes*, which seems likely that caffeine has its unique effect on the intestinal microbiome. *Bacteroidetes* is a Gram-negative bacterium containing LPS, a potent activator of toll-like receptor 4(TLR4), which causes inflammatory responses and cytokine expression and secretion. In addition, Lower *Bacteroidetes* may be associated with lower *Bacteroides* at the genus level. *Bacteroides, Lactobacillus* and *Lactococcus* containing bile salt hydrolase (BSH) can hydrolyze conjugated bile acids into unconjugated bile acids [37]. It has been reported that conjugated bile acid is a nuclear farnesoid X receptor (FXR) antagonist [33]. Decreased BSH leads to increased conjugated bile acids, which increases the conversion of cholesterol to bile acids and reduces lipid accumulation. Likewise, in our plasma data, cholic acid, which was elevated in insulin-resistant mice and humans [9, 34], was decreased by caffeine, while glycocholic acid was increased. This may suggest that the improvement of dyslipidemia by caffeine may be related to bile acid metabolism. *Bifidobacterium* is a probiotic. In a human report, 16 healthy subjects drank a daily dose of 3 cups of coffee for three weeks, which raised the number of *Bifidobacterium* compared with human faeces before coffee consumption [35]. This bifidogenic effect of coffee was also discovered in animal models [32]. Meanwhile, caffeine also increased this probiotic in the NAFLD patients [36]. In accordance with our results, which hint the bifidogenic effect of coffee may be related to caffeine. Moreover, *Bifidobacterium* and *Ruminococcaceae* are short-chain fatty acid (SCFAs)-producing bacteria; SCFAs have been shown to increase gut barrier function [37] and stimulate pancreatic islet beta-cell growth and proliferation, regulate the body's insulin sensitivity [38]. *Desulfovibrio*, significantly elevated by caffeine, was shown to produce acetic acid and regulate liver lipid metabolism in mice to alleviate non-alcoholic fatty liver disease [39]; to produce H2S, which maintains lipid metabolism balance [40] and reduces systemic inflammation [41]. In addition, the present study also shown *Bifidobacterium*, *Ruminococcaceae* and *Desulfovibrio* were negatively associated with obesity indicators.

Serum metabolomics showed that the serum metabolites of the three groups were separated from each other. However, caffeine did not move the plasma metabolites of HFD mice closer to that of NCD mice, which may be the effect of caffeine as a foreign substance in the metabolism. Elevations of caffeine metabolites such as 1,7-Dimethylxanthine in plasma may illustrate this point. In our data, 1,7-Dimethylxanthine was positively correlated with *Dubosiella*, *Alkaninndiges* and *Faecalibaculum*, possibly indicating that changes in the intestinal microbiome are associated with caffeine metabolism. *Dubosiella* significantly elevated in response to caffeine, is not yet well studied. Still, this bacterium was negatively correlated with most inflammatory factors and indicators of obesity and positively associated with butyric acid [42, 43]. Moreover, *Dubosiella* is positively correlated with Adenosine 5'-monophosphate (AMP) and Linoleic acid, which Implies the relationship between *Dubosiella* and energy metabolism. Metabolic pathway enrichment analysis showed that caffeine could regulate Steroid hormone biosynthesis, Primary bile acid biosynthesis, Biosynthesis of unsaturated fatty acids, Glycine, serine and threonine metabolism, Linoleic acid metabolism, Pyruvate metabolism and so on. The Biosynthesis of steroid hormones, the Biosynthesis of primary bile acids and the Biosynthesis of unsaturated fatty acids are closely related to lipid metabolism. Disturbances in lipid metabolism often accompany insulin resistance. In a study of the activity and function of the cytochrome P450 side-chain cleavage enzyme (CYP11A1), the rate-limiting enzyme for the conversion of cholesterol to pregnenolone, it was found that incubation of isolated mitochondria with the cholesterol analogue 22R-hydroxycholesterol resulted in the efficient formation of pregnenolone, the direct precursor for the synthesis of all steroid hormones [44]. Estrogen has been reported to increase insulin sensitivity [45]. Similarly, 22R-hydroxycholesterol is a natural ligand for the liver X receptor [LXR], and activation of LXR improves TNFα-induced insulin resistance in brown adipocytes [46]. All of the above suggests that the improvement of insulin resistance by caffeine may be related to steroid hormone synthesis. Glycine, serine, threonine, linoleic acid and pyruvate acid ultimately produce energy metabolism through the tricarboxylic acid cycle. Caffeine is known to activate beta receptors, increase plasma cAMP concentrations and promote lipolysis and beta-oxidation [47].

## Conclusion

This study illustrates the ameliorative effect of caffeine on diet-induced obesity mice by combining 16S rRNA Sequencing and plasma metabolomics. Caffeine modulates plasma lipid metabolism disorders, ameliorates insulin resistance, and may change the gut microbial composition by promoting beneficial bacteria and reducing harmful bacteria. In addition, serum metabolomics suggests that caffeine may act by regulating bile acid metabolism, lipid metabolism and energy metabolism. The present study is the first article that caffeine can affect the gut microbiota and provides a possible mechanism of intestinal microbiota to account for the effect of caffeine on insulin resistance.

## Data Availability

Data are available upon reasonable request.
